# Therapeutic potential of anti-IL-6 therapies for granulocytic airway inflammation in asthma

**DOI:** 10.1186/s13223-015-0081-1

**Published:** 2015-04-12

**Authors:** Derek K Chu, Amal Al-Garawi, Alba Llop-Guevara, Regina A Pillai, Katherine Radford, Pamela Shen, Tina D Walker, Susanna Goncharova, William J Calhoun, Parameswaran Nair, Manel Jordana

**Affiliations:** Department of Pathology & Molecular Medicine, McMaster Immunology Research Centre, Hamilton, ON Canada; Division of Respirology, Department of Medicine, McMaster University, Hamilton, Ontario Canada; Division of Pulmonary and Critical Care Medicine, and Institute for Translational Sciences, University of Texas Medical Branch, Galveston, Texas USA

**Keywords:** Airway inflammation, Asthma, Allergy, Bronchitis, Eosinophil, Neutrophil, Granulocyte, IL-6, IL-6R, House dust-mite (HDM)

## Abstract

**Background:**

Determining the cellular and molecular phenotypes of inflammation in asthma can identify patient populations that may best benefit from targeted therapies. Although elevated IL-6 and polymorphisms in IL-6 signalling are associated with lung dysfunction in asthma, it remains unknown if elevated IL-6 levels are associated with a specific cellular inflammatory phenotype, and how IL-6 blockade might impact such inflammatory responses.

**Methods:**

Patients undergoing exacerbations of asthma were phenotyped according to their airway inflammatory characteristics (normal cell count, eosinophilic, neutrophilic, mixed granulocytic), sputum cytokine profiles, and lung function. Mice were exposed to the common allergen, house dust-mite (HDM), in the presence or absence of endogenous IL-6. The intensity and nature of lung inflammation, and levels of pro-granulocytic cytokines and chemokines under these conditions were analyzed.

**Results:**

Elevated IL-6 was associated with a lower FEV_1_ in patients with mixed eosinophilic-neutrophilic bronchitis. In mice, allergen exposure increased lung IL-6 and IL-6 was produced by dendritic cells and alveolar macrophages. Loss-of-function of IL-6 signalling (knockout or antibody-mediated neutralization) abrogated elevations of eosinophil and neutrophil recruiting cytokines/chemokines and allergen-induced airway inflammation in mice.

**Conclusions:**

We demonstrate the association of pleiotropic cellular airway inflammation with IL-6 using human and animal data. These data suggest that exacerbations of asthma, particularly those with a combined eosinophilic and neutrophilic bronchitis, may respond to therapies targeting the IL-6 pathway and therefore, provide a rational basis for initiation of clinical trials to evaluate this.

## Background

Asthma is a chronic disease of the airways characterized by reversible airflow obstruction, airway hyperresponsiveness, and airway inflammation. While these are disease defining features, asthma has more recently been recognized as a widely heterogeneous disease with multiple clinical variants, subtypes depending on factors such as severity, responsiveness to corticosteroids, or nature of airway inflammation. In regards to the latter, airway inflammometry has emerged as a critical consideration in the control of asthma and design of novel, targeted therapeutics tailored to the specific composition of airway cellular inflammatory infiltrate [1-3]. For example, we have shown that the selection of patients with an eosinophilic asthmatic phenotype is critical for the efficacy of anti-IL-5 therapy in controlling asthma exacerbations [4]. Thus, determining the cellular and molecular phenotypes of inflammation in asthma can identify patient populations that may best benefit from targeted therapies.

IL-6 is a pleiotropic cytokine that can be produced by many cell types in response to a wide array of inflammatory stimuli and cytokines [5]. IL-6 binds soluble or membrane-bound receptors (sIL-6R or mIL-6R, respectively), before associating with and signalling through gp130, a ubiquitously expressed trans-membrane protein [5].

As recently reviewed [6], IL-6 and sIL-6R have consistently been observed to be elevated in the airways of children and adults with asthma, with levels of these proteins being directly correlated with disease severity, and inversely correlated with forced expiratory volume in 1 second (FEV_1_) [7-9]. Immunohistochemistry (IHC) of endobronchial biopsies from patients with asthma showed that IL-6R was mainly expressed by airway epithelium, smooth muscle, and vascular endothelium. IHC of bronchoalveolar lavage (BAL) from the same subjects showed that IL-6 was expressed by macrophages and granulocytes [10]. IL-6 and sIL-6R have also been reported to be elevated in serum of patients with asthma [11]. Further, recent genome-wide association studies have identified polymorphisms in IL-6R as novel asthma risk loci that correlate with lower percent predicted FEV_1_, forced vital capacity (FVC) and FEV_1_/FVC ratio [10,12]. Such polymorphisms have been predicted to enhance proteolytic cleavage of IL-6R from cell surfaces, thereby increasing levels of sIL-6R and subsequent signalling through IL-6- sIL-6R-gp130 complexes.

Although the cellular and molecular phenotypes of inflammation in asthma can identify patient populations that may best benefit from targeted therapies, mepolizumab for eosinophilic-asthma for example [4], an association between IL-6 and a specific airway inflammatory phenotype of asthma is not known. Here, we show that IL-6 is associated with mixed eosinophilic-neutrophilic bronchitis during exacerbations of asthma and worse pulmonary function in humans. Using a well-established animal model of asthma, we test the hypothesis that IL-6 drives granulocytic inflammation in the lungs. Indeed, in the absence of IL-6 allergen-induced allergic airway inflammation is abrogated. Thus, exacerbations of asthma, particularly those with a combined eosinophilic and neutrophilic bronchitis, may respond to therapies targeting the IL-6 pathway and therefore, these data provide a rational basis for initiation of clinical trials to evaluate this.

## Methods

### Study subjects and specimen collection and analysis

Induced sputum was collected from consecutive patients with physician-diagnosed asthma self-reporting exacerbations (defined as increased cough, wheeze or sputum production for longer than 2 days and whose Asthma Control Questionnaire [13] score had changed by more than 0.5 units) attending the Firestone Institute for Respiratory Health outpatient clinic (Hamilton, Ontario). A diagnosis of asthma was based on a compatible clinical history with evidence of reversible airflow limitation (increase in FEV_1_ of 15% or greater following a bronchodilator) or airway hyper-responsiveness (provocative concentration of methacholine causing a 20% fall in FEV_1_). Sputum induction and examination of cytokines and total and differential cell counts were performed as described previously [14]. Sputum cytokines were quantitated using a Luminex assay (Luminex, Austin, TX). Spirometry was performed according to the standards of the American Thoracic Society. The study was approved by the Research Ethics Board of St Joseph’s Healthcare, Hamilton, Ontario. All patients provided written informed consent.

### Animal model of asthma

Age, sex, vendor and strain-matched controls were used in all experiments. Wild-type (WT) and IL-6 KO (B6.129S2-*Il6*^*tm1Kopf*^*/*J) mice were from JAX laboratories (Bar Harbor, Maine). Some groups of WT mice received either anti-mouse IL-6 (MP5-20 F3, R&D systems or BioXCell) or control Rat IgG (Sigma). As previously described [15,16], mice received 25 μg house dust-mite (HDM, Dermatophagoides pteronyssinus, Greer Laboratories) in 10 μl saline intranasally (i.n.) once daily for 10 consecutive days. Control mice received saline only i.n. 24 h later, the lungs were extracted and subjected to BAL twice (0.25 ml followed by 0.2 ml) with PBS containing COMPLETE protease inhibitor (Roche), and approximately 0.25 – 0.3 ml of the instilled fluid was retrieved consistently. Total cell counts were then determined using a hemocytometer. Each BAL sample was then centrifuged and the supernatants collected and stored at -20°C. Cytospins were prepared and stained with Protocol Hema 3 set (Fisher Scientific) and, 500 cells were counted and identified as monocytes, lymphocytes, neutrophils and eosinophils using standard hemocytological criteria. In some experiments, the lungs were dissected without BAL collection and placed in PBS with COMPLETE protease inhibitor at 4°C for tissue homogenate preparation. Alternatively, lungs were perfused with PBS and kept in ice-cold HBSS until processing for flow cytometric analysis. All procedures were approved by the McMaster University Research Ethics Board.

### Lung tissue homogenates and analysis

Whole lungs were homogenized in 1.5 ml PBS supplemented with COMPLETE protease inhibitor (Roche, Laval, QC, Canada). After homogenization, 150 μl of 10% Triton X-100 was added and samples were rocked at 4°C for 1 h. The supernatant was collected following a 15 min spin at 12,000 rpm at 4°C and stored at -70°C. Cytokines were measured in lung homogenates using Luminex 100 Total System and kits from from Upstate (Charlottesville, VA).

### Dendritic cells and macrophages

As previously reported [17], granulocyte-macrophage-colony stimulating factor (GM-CSF)–derived dendritic cells were generated and then incubated for 24 h with media, HDM, *Escherichia coli* 0111:B4 LPS (Cell culture tested; Sigma) or 25 multiplicity of infection replication deficient vesicular stomatitis virus (VSV)-∆M51, which transduces dendritic cells without significant progeny virus production or effect on viability. As previously reported [18], alveolar macrophages were recovered from the lungs naïve mice with ice cold PBS supplemented with 0.5 mM EDTA and, then, washed with complete RPMI. 5.22 × 10^5^ macrophages per well were plated and allowed to adhere at 37°C for 1 h. Non-adherent cells were removed by gently washing three times with warm PBS. Macrophages were stimulated for 24 h with media, HDM, or LPS. Cell-free supernatants were analyzed for IL-6 by ELISA (R&D).

### Lung cell isolation and flow cytometric analysis

As described previously [15], total lung cells were isolated by collagenase digestion (Collagenase type I; Life Technologies, Burlington, ON, Canada), washed twice in PBS with 0.5% bovine serum albumin, and then filtered through 40-μm cell strainer.

In all assays, cells were incubated with anti-FcγRII/IIIb before incubation with fluorochrome-conjugated antibodies, dead cells excluded by propidium iodide (Sigma) uptake and gated on singlets. Antibodies used were from eBioscience, BD Biosciences, Invitrogen, or Biolegend and pre-titrated to determine optimal concentration: CD45-allophyocyannin (APC)-Cyanine(Cy)7, CD3-Pacific Blue, CD11c-fluorescein isothiocyanate (FITC), B220-phycoerythrin (PE)-Cy5, major histocompatibility complex II-Alexa Fluor 700, Siglec-F-PE, Ly6C-Peridinin Chlorophyll Protein Complex (PerCP)-Cy5.5, DX5-PE-Cy7, CD4-APC, Gr-1-Pacific Orange. Fluorescence minus one controls were used for gating. Data were collected using an LSRII (BD Biosciences) and analyzed using FlowJo software (Tree Star). More than 300,000 events were collected for each sample.

### Statistics

Comparisons were made using unpaired t-tests or one-way ANOVA. p < 0.05 was considered statistically significant.

## Results and discussion

### IL-6 associates with mixed eosinophilic-neutrophilic exacerbations of asthma and worse pulmonary function in humans

To explore the impact of IL-6 in asthma, we first analyzed induced sputum samples [14] from patients with asthma with different cellular phenotypes in their sputum. In our clinic, sputum is collected at the time of exacerbation in the routine clinical management of patients with moderate to severe asthma in order to assess bronchitis to optimize the anti-inflammatory treatment. The samples reported in this study were from consecutive patients who had self-reported exacerbations (defined as increased cough, wheeze or sputum production for longer than 2 days and whose Asthma Control Questionnaire score had changed by more than 0.5 units) who attended the clinic during the study period.

**Figure 1:**
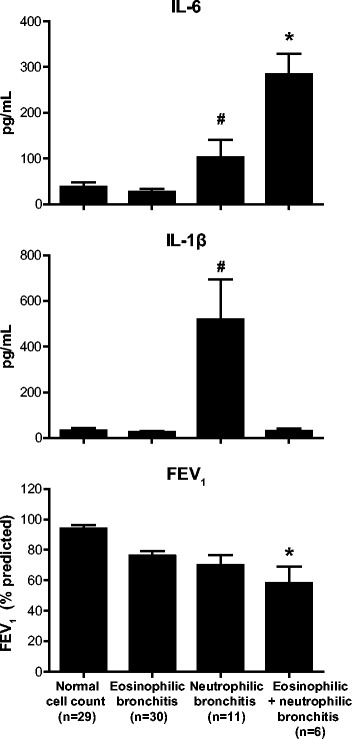
IL-6 associates with eosinophilic-neutrophilic granulocytic inflammation and worse pulmonary function in patients with asthma. Sputum IL-6 levels (upper panel) were highest and FEV_1_ (% predicted) (lower panel) was lowest in patients with asthma who had a combined eosinophilic and neutrophilic bronchitis compared to patients with asthma who had normal cell counts in sputum, an eosinophilic bronchitis (>3%) or a neutrophilic bronchitis (total cell count >10^6^/g and neutrophils >65%). IL-1β was elevated only in the neutrophilic bronchitis group (middle panel). Mean + SEM. Sample size shown in lower panel. *p < 0.05 vs Neutrophilic bronchitis and eosinophilic bronchitis groups. ^#^p < 0.05 vs eosinophilic bronchitis group.

Using Luminex bead-based multiplex immunoassay of dithiothreitol (DTT)-treated sputum supernatants, we found significantly higher levels of IL-6 in patients with asthma with a mixed eosinophilic and neutrophilic bronchitis compared to patients with asthma with an isolated eosinophilic (total cell count <10 × 10^6^/g, eosinophil >3%) or intense neutrophilic bronchitis (total cell count > 15 × 10^6^/g, neutrophils >65%) or normal cell counts (Figure 1). In contrast, patients with isolated neutrophilic bronchitis had elevated levels of sputum IL-1β, which has been associated with bacterial exacerbation of obstructive airway diseases [19,20]. TNF-ɑ was below the limit of detection in the majority of patients (not shown), and clinical trials have generated conflicting results with anti-TNF-based therapies for asthma [2]. In terms of lung function, patients with mixed eosinophilic/neutrophilic bronchitis had the greatest degree of airflow obstruction compared to the other three groups (Figure 1). Analysis of variance across groups revealed no statistical difference in doses of corticosteroids used at time of sample collection (median dose of ICS 1500 μg in the eosinophilic group, 1000 μg in the 2 other groups). Thus, sputum IL-6 is associated with mixed eosinophilic/neutrophilic asthmatic airway inflammation and impaired lung function.

### IL-6 is produced by allergen-stimulated mononuclear phagocytes and mediates granulocytic allergic airway inflammation through elicitation of eosinophil and neutrophil chemokines and cytokines in mice

**Figure 2:**
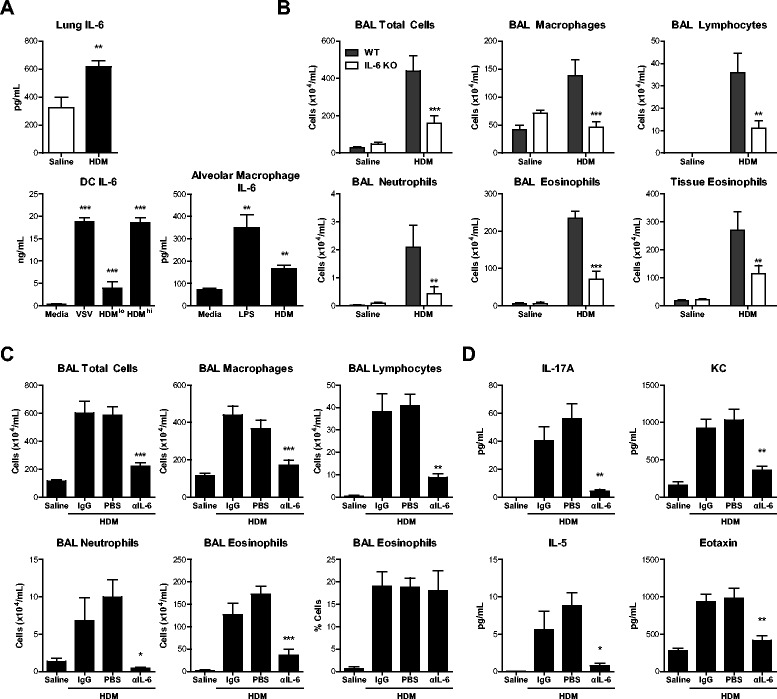
IL-6 is produced by allergen-stimulated mononuclear phagocytes and mediates allergic airway inflammation through eosinophil/neutrophil chemokines/cytokines. WT or IL-6 KO mice received 25 μg HDM i.n. daily for 10d, with or without 50 μg anti-IL-6 (αIL-6) or control IgG i.p. on d0, 3, 5 and 7. (**A**) IL-6 by ELISA in lung homogenates, or cultures of dendritic cells or alveolar macrophages. (**B, C**) Cell counts from bronchoalveolar lavage (BAL) or digested lung tissue quantified by hemocytometer manual counting with Turks, followed by differential cell counting of Hema 3-stained cytospins or flow cytometry. (**D**) Cytokines in lung tissue homogenates quantified by Luminex. Mean + SEM, n = 3-5 mice per group per experiment, 2-3 independent experiments performed. *p < 0.05 compared to saline, media, WT HDM, or HDM + IgG control groups. **p < 0.01. ***p < 0.001.

To investigate a causative role for IL-6 in asthma we used a well-established mouse model of HDM-induced allergic airway inflammation, where sensitization and inflammation are achieved by the i.n. administration of allergen without any exogenous adjuvant [16,21]. Mice exposed to HDM in this manner exhibited elevated levels of IL-6 in the lung (Figure 2A), suggesting that IL-6 drives inflammatory responses to HDM. As mononuclear phagocytes have been proposed to be a major source of IL-6 [10], we questioned if HDM elicited IL-6 from such cells *in vitro*. Indeed, compared to media-exposed dendritic cells, HDM induced IL-6 production in a dose dependent manner (Figure 2A). Likewise, and as previously shown [18], alveolar macrophages exposed to HDM *ex vivo* produced IL-6 (Figure 2A). VSV and LPS was used as positive controls for eliciting IL-6 production [17,18]. Thus, HDM-induced airway inflammation is associated with IL-6 production, at least from mononuclear phagocytes.

We next evaluated HDM-induced immune responses in the absence of IL-6 *in vivo*. Compared to WT mice exposed to HDM alone or along with control IgG, we found that IL-6 KO mice (Figure 2B) or WT mice (both C57BL/6 background from Jackson Laboratories) treated with 50 μg of anti-IL-6 antibody i.p. (Figure 2C) mounted significantly impaired airway inflammatory responses. Differential cell counts of BAL cells and flow cytometry of isolated lung cells revealed global impairment in inflammation, i.e. reduction in all cell types, in both the airway and tissue (Figure 2B, bottom right). This reduced inflammatory response was broad based, as the number, but not percentage, of eosinophils was decreased between allergic mice treated with control IgG or anti-IL-6 (Figure 2C, bottom right). Notably, inhibition of neutrophilic and eosinophilic inflammation was associated with markedly lower activating and chemotactic cytokines in the lung, IL-17A and KC, and IL-5 and eotaxin, respectively (Figure 2D). Thus, IL-6, at least derived from dendritic cells and alveolar macrophages, promotes allergen-induced airway inflammation through pro-granulocytic cytokine and chemokine production.

## Conclusions

Clinical data show that IL-6/IL-6R is differentially expressed in asthmatics versus healthy controls, and that this is associated with airway dysfunction [6]. IL-6R polymorphisms may contribute to asthma severity [6]. Here, we observed high levels of IL-6 in the sputum of patients with mixed eosinophilic/neutrophilic airway inflammation and that this was associated with worse pulmonary function. Our preclinical data demonstrate that IL-6 is elevated in the lung during airway inflammation, and that inhibition of IL-6 signalling decreases asthmatic inflammatory responses irrespective of whether that inflammation is neutrophilic, eosinophilic, or mixed, through downregulation of granulocyte activating and recruiting cytokines and chemokines. Previous studies using surrogate allergen systems with intraperitoneal, rather than mucosal sensitization, have led to inconsistent results, in part due to exclusive examination of either IL-6 KO mice or WT mice treated with anti-IL-6 antibodies [5,22]. Here, we used a common environmental allergen, HDM, in a model involving only mucosal exposure. Further, we utilized both KO as well as antibody neutralization strategies to generate consistent results showing that IL-6 drives HDM-induced airway inflammation. Thus, our clinical and preclinical data suggests that patients with asthma, such as those with mixed-eosinophilic/neutrophilic bronchitis selected through inflammometry, may benefit from therapies targeting the IL-6 pathway. Altogether, these data provide a rational basis to initiate clinical trials of anti-IL-6 based therapies for patients with asthma.

## Abbreviations

FEV_1_: Forced expiratory volume in 1 second
FVC: Forced vital capacity
IHC: Immunohistochemistry
BAL: Bronchoalveolar lavage
FVC: Forced vital capacity
DTT: Dithiothreitol
HDM: House dust-mite
KO: Knockout
WT: Wild-type
KC: Keratinocyte-derived chemokine
i.n: Intranasal
GM-CSF: Granulocyte-macrophage-colony stimulating factor
VSV: Vesicular stomatitis virus
APC: Allophycocyanin
Cy: Cyanine
FITC: Fluorescein isothiocyanate
PerCP: Peridinin chlorophyll protein complex

## References

[1] Nair P (2013). Update on clinical inflammometry for the management of airway diseases. Can Respir J.

[2] Dasgupta A, Neighbour H, Nair P (2013). Targeted therapy of bronchitis in obstructive airway diseases. Pharmacol Ther.

[3] Nair P, Dasgupta A, Brightling CE, Chung KF (2012). How to diagnose and phenotype asthma. Clin Chest Med.

[4] Nair P, Pizzichini MM, Kjarsgaard M, Inman MD, Efthimiadis A, Pizzichini E (2009). Mepolizumab for prednisone-dependent asthma with sputum eosinophilia. N Engl J Med.

[5] Rincon M (2012). Interleukin-6: from an inflammatory marker to a target for inflammatory diseases. Trends Immunol.

[6] Rincon M, Irvin CG (2012). Role of IL-6 in asthma and other inflammatory pulmonary diseases. Int J Biol Sci.

[7] Fitzpatrick AM, Higgins M, Holguin F, Brown LA, Teague WG (2010). The molecular phenotype of severe asthma in children. J Allergy Clin Immunol.

[8] Morjaria JB, Babu KS, Vijayanand P, Chauhan AJ, Davies DE, Holgate ST (2011). Sputum IL-6 concentrations in severe asthma and its relationship with FEV1. Thorax.

[9] Neveu WA, Allard JL, Raymond DM, Bourassa LM, Burns SM, Bunn JY (2010). Elevation of IL-6 in the allergic asthmatic airway is independent of inflammation but associates with loss of central airway function. Respir Res.

[10] Hawkins GA, Robinson MB, Hastie AT, Li X, Li H, Moore WC (2012). The IL6R variation Asp(358)Ala is a potential modifier of lung function in subjects with asthma. J Allergy Clin Immunol.

[11] Yokoyama A, Kohno N, Sakai K, Kondo K, Hirasawa Y, Hiwada K (1997). Circulating levels of soluble interleukin-6 receptor in patients with bronchial asthma. Am J Respir Crit Care Med.

[12] Ferreira MA, Matheson MC, Duffy DL, Marks GB, Hui J, Le Souef P (2011). Identification of IL6R and chromosome 11q13.5 as risk loci for asthma. Lancet.

[13] Juniper EF, O’Byrne PM, Guyatt GH, Ferrie PJ, King DR (1999). Development and validation of a questionnaire to measure asthma control. Eur Respir J.

[14] Pizzichini E, Pizzichini MM, Efthimiadis A, Hargreave FE, Dolovich J (1996). Measurement of inflammatory indices in induced sputum: effects of selection of sputum to minimize salivary contamination. Eur Respir J.

[15] Al-Garawi AA, Fattouh R, Walker TD, Jamula EB, Botelho F, Goncharova S (2009). Acute, but not resolved, influenza A infection enhances susceptibility to house dust mite-induced allergic disease. J Immunol.

[16] Cates EC, Fattouh R, Wattie J, Inman MD, Goncharova S, Coyle AJ (2004). Intranasal exposure of mice to house dust mite elicits allergic airway inflammation via a GM-CSF-mediated mechanism. J Immunol.

[17] Chu DK, Llop-Guevara A, Walker TD, Flader K, Goncharova S, Boudreau JE (2013). IL-33, but not thymic stromal lymphopoietin or IL-25, is central to mite and peanut allergic sensitization. J Allergy Clin Immunol.

[18] Llop-Guevara A, Chu DK, Walker TD, Goncharova S, Fattouh R, Silver JS (2014). A GM-CSF/IL-33 pathway facilitates allergic airway responses to sub-threshold house dust mite exposure. PLoS One.

[19] Barker BL, Haldar K, Patel H, Pavord ID, Barer MR, Brightling CE (2015). Association Between Pathogens Detected Using Quantitative Polymerase Chain Reaction With Airway Inflammation in COPD at Stable State and Exacerbations. Chest.

[20] Bafadhel M, McKenna S, Terry S, Mistry V, Reid C, Haldar P (2011). Acute exacerbations of chronic obstructive pulmonary disease: identification of biologic clusters and their biomarkers. Am J Respir Crit Care Med.

[21] Cates EC, Fattouh R, Johnson JR, Llop-Guevara A, Jordana M (2007). Modeling responses to respiratory house dust mite exposure. Contrib Microbiol.

[22] Neveu WA, Allard JB, Dienz O, Wargo MJ, Ciliberto G, Whittaker LA (2009). IL-6 is required for airway mucus production induced by inhaled fungal allergens. J Immunol.

